# The Power of Iodide of Potassium in Expediting Mercurial Salivation

**Published:** 1860-03

**Authors:** Bernard Kelly

**Affiliations:** Physician to the New York Dispensary


*The Power of the Iodide of Potassium in Expediting Mercurial Salivation.* By BERNARD KELLY, M.D., Physician to the New York Dispensary.

Most physicians, in extensive practice, must have from time to time observed that there are many patients, who, from some unknown idiosyncrasy, obstinately resist the action of mercury for a long-protracted period, or even indefinitely. Hence the various expedients adopted by them for the purpose of bringing the refractory system under its influence. Some employ the endermic and iatroleptic modes; some fumigations; and others, the introduction of blue ointment within the rectum. These several methods rarely fail when tried separately, or in succession. All of them, however, are attended with no inconsiderable amount of labor, loss of time, and indelicate exposure, which last (in case of females being subjects of treatment) involves no trivial obstacle to their indiscriminate employment.

We have never failed to produce the specific action of mercury, in a remarkably short space of time, by the simultaneous use of large doses of the iodide of potassium. This method possesses many peculiar advantages over those already mentioned. In the first place, it is expeditious and quite manageable, incurring neither unnecessary labor nor exposure; and secondly, the salivation induced by their combined action is fully as efficacious as that produced by mercury alone; with the additional advantage, that the ptyalism, brought on by the assistance of the iodide, is always milder; never producing the horrible fœtor and sloughing so characteristic of the former agent, and usually subsides within the space of a few days. These considerations are very important, in a practical point of view, when nothing short of salivation is to be relied upon in the treatment of a grave disease; or when our patient, as it often happens, is an aged or debilitated subject, or one in whom the depressing effects of mercury are to be cautiously guarded against. By this means we can graduate, as it were, the precise amount of ptyalism to be induced in any given case; and also, when the urgency of the symptoms demands a prompt and decided action, greatly diminish the length of time necessary to bring about the desired result.

In treating infantile diseases, both substances should never, in our opinion, be given simultaneously. We remember one case, in particular, where a dangerous salivation, which all but terminated fatally, had been induced by a few doses of calomel, a solution of the iodide of potassium being administered at the same time. The little patient had been suffering from an acute attack of meningitis, but ultimately recovered from the complication of both affections—thanks to the fortunate services rendered by the internal and topical employment of quinine and the chlorate of potash. The *nimia cura medici*, which is always a source of error to the practitioner, and mischief, if not death, to his patients, becomes doubly so when active agents are blindly pressed, through an overweening solicitude to heal. The well-known difficulty of inducing salivation in the child by the ordinary means, lends additional testimony, through the example cited, of the facility with which mercury exerts its specific action upon the human system, when aided by its great rival and frequent substitute—the iodide of potassium.

This fact, as far as we are aware, has not been broached, as yet, by any of our professional brethren; and, therefore, we shall feel amply gratified should its knowledge prove an effectual means to check the rapid course of a formidable disease; or serve as a beacon to warn them in time of the shoals and quicksands on which, unconsciously, they may happen to be rushing.

